# 260. The Unfortunate Consequence of Immunosuppression in a Renal Transplant Patient

**DOI:** 10.1093/ofid/ofab466.462

**Published:** 2021-12-04

**Authors:** Franklin Mikell, Rabindra Ghimire

**Affiliations:** 1 Vidant Health, Greenville, North Carolina; 2 East Carolina University, Greenville, NC

## Abstract

**Background:**

Nocardia is a slow-growing aerobic-actinomycete that belongs to the family Nocardiaceae. Major predisposing factors include corticosteroid use, organ transplantation, low CD4 count, and hematologic malignancies. The most commonly affected organs are lungs, mainly via inhalation; however, the most common extrapulmonary site is central nervous system.

**Methods:**

Matrix Assisted Laser Desorption Ionization - Time of Flight (MALDI-ToF) or 16srRNA sequencing are more reliable methodologies for accurate identification of *Nocardia* to the species level. To our knowledge, our patient represented the first U.S. case of *N. bejingensis* opportunistic disseminated infection in a renal transplant patient although similar cases have been previously reported outside the U.S.

GMS stain

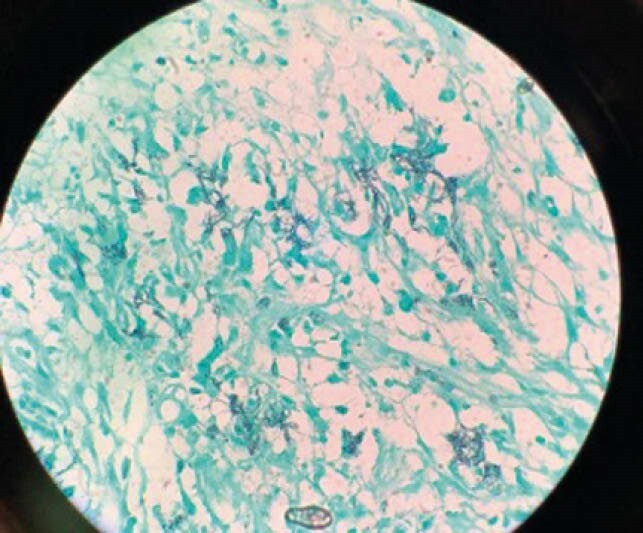

Gram Stain of Nocardia

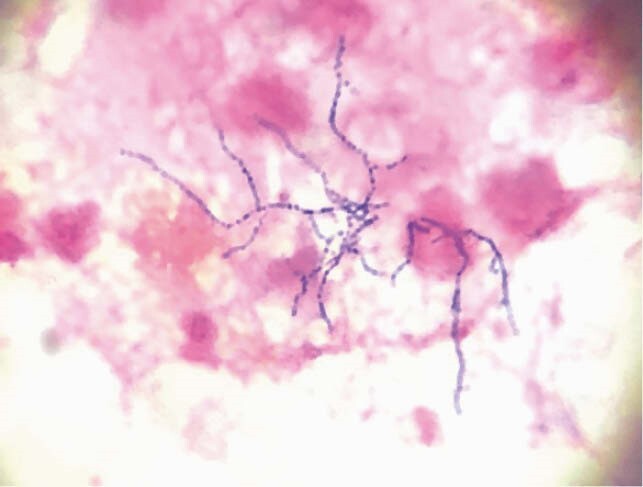

**Results:**

We present a 31-year-old Caucasian male status post renal transplant four years ago on immunosuppressants with left arm myoclonic jerks. In addition, there was an associated unilateral left frontal headache of four to five day duration. His chest CT revealed consolidative process in the right lower lobe and pleural effusion. MRI of the brain revealed multiple ring-enhancing lesions. Patient underwent left frontal craniotomy with resection and a complete evacuation of brain abscess. His brain abscess and pleural fluid cultures revealed Gram positive rods, which were subsequently identified as *Nocardia beijingensis* by MALDI-TOF and confirmed by 16srRNA sequencing. He was treated with intravenous imipenem & trimethoprim – sulfamethoxazole with subsequent clinical improvement.

MRI Brain w/ contrast

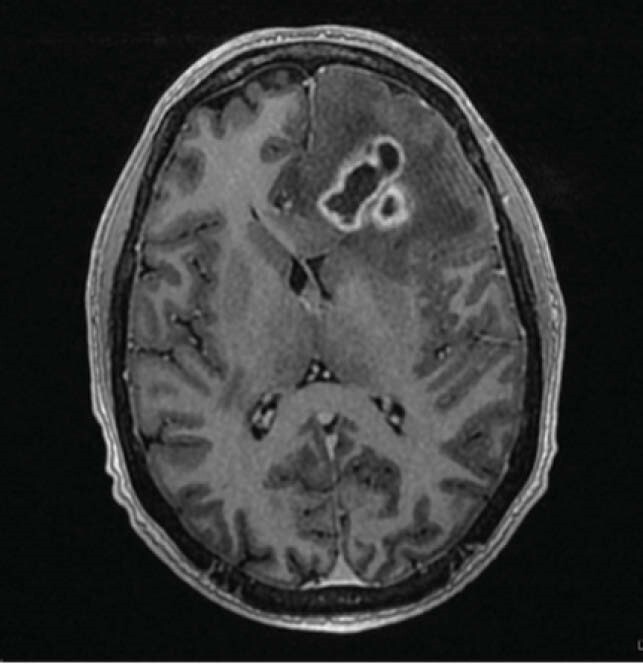

Head CT s/p left frontal craniotomy with resection & evacuation of abscess

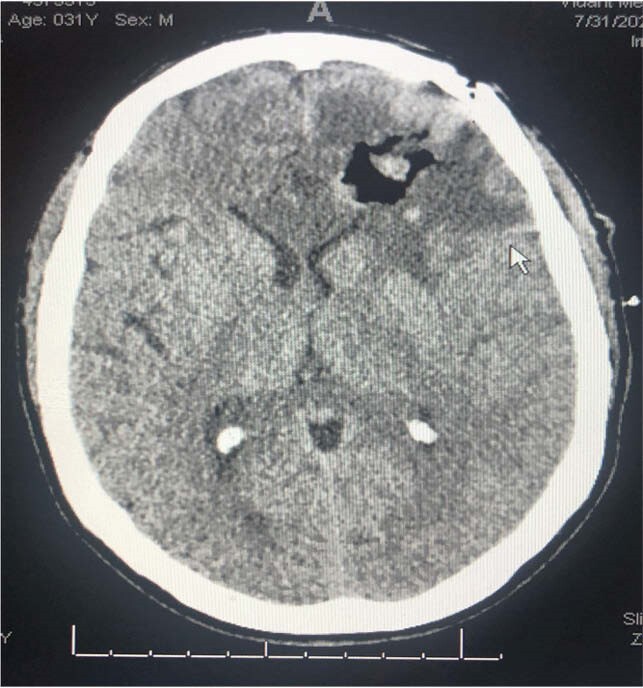

Chest CT

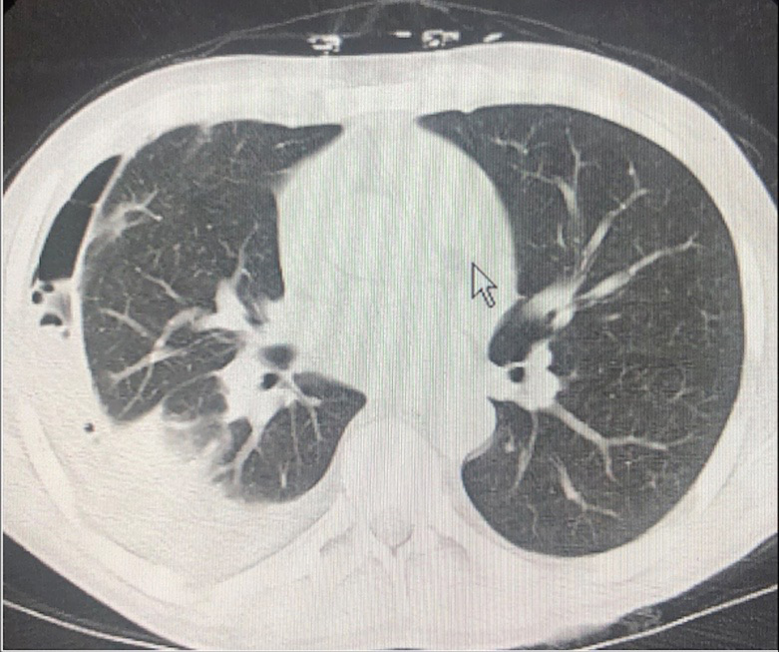

**Conclusion:**

Different *Nocardia* species have a wide geographic distribution with varying pathogenic traits, and antimicrobial susceptibility. Hence, the identification of the specific species of *Nocardia* is crucial to provide a proficient level of patient care. *Nocardia bejingensis* is a newly discovered species of *Nocardia* that was first isolated in 2001 in China. Only six cases of *N. beijingensis* affecting CNS have been reported up to date in the United States. It is unclear of the geographic distribution and variable antimicrobial susceptibility of *Nocardia bejingensis* but we can confirm the first reported case of an opportunistic disseminated infection in a renal transplant patient in the United States.

Agar

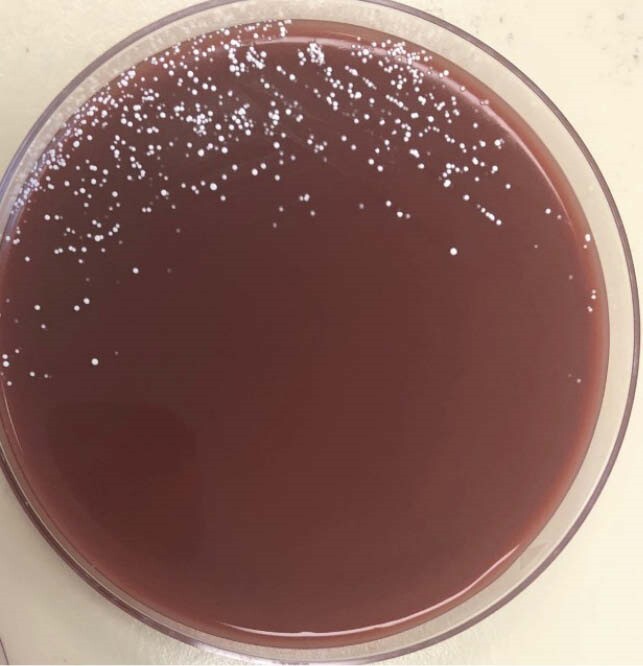

**Disclosures:**

**All Authors**: No reported disclosures

